# Peritumoral epilepsy: Relating form and function for surgical success

**DOI:** 10.1016/j.yebeh.2014.05.009

**Published:** 2014-09

**Authors:** Christopher J.A. Cowie, Mark O. Cunningham

**Affiliations:** a Department of Neurosurgery, Royal Victoria Infirmary, Queen Victoria Road, Newcastle upon Tyne, UK; b Institute of Neuroscience, The Medical School, Framlington Place, Newcastle University, Newcastle upon Tyne, UK

**Keywords:** Tumor, Epilepsy, Oscillations, Microcircuit, Neocortex

## Abstract

Seizures are a prominent symptom in patients with both primary and secondary brain tumors. Medical management of seizure control in this patient group is problematic as the mechanisms linking tumorigenesis and epileptogenesis are poorly understood. It is possible that several mechanisms contribute to tumor-associated epileptic zone formation. In this review, we discuss key candidates that may be implicated in peritumoral epileptogenesis and, in so doing, hope to highlight areas for future research. Furthermore, we summarize the current role of antiepileptic medications in this type of epilepsy and examine the changes in surgical practice which may lead to improved seizure rates after tumor surgery. Lastly, we speculate on possible future preoperative and intraoperative considerations for improving seizure control after tumor resection.

**This article is part of a Special Issue entitled “NEWroscience 2013”.**

## Highlights

• Tumor-associated epilepsy has a significant detrimental impact on patients.
• Low-grade gliomas more commonly give rise to seizures.
• The peritumoral region is likely to contain the epileptogenic zone.
• Multiple mechanisms contribute to the peritumoral epileptogenic microcircuit.
• Enhanced image-guided resection of the peritumoral zone may improve outcomes.

## 1 Introduction

Tumor-associated epilepsy (TAE) is a debilitating condition, causing distress and adversely affecting the quality of life of those suffering from brain tumors [1], [2], [3]. Furthermore, in patients who have had tumor surgery, both the presence of postoperative seizures and the antiepileptic medication used to treat them have been shown to have a detrimental neuropsychological effect [2]. In rare cases, TAE can be even more devastating, giving rise to sudden unexpected death [4]. Despite its major clinical and social impact, the underlying pathophysiological causes of TAE are poorly understood, and, as a result, its treatments, both pharmacological and surgical, are of limited efficacy. Epilepsy associated with tumors has been shown to have a greater refractivity to antiepileptic drug treatments, and, in those who have had surgery for their tumor, seizures may persist postoperatively [5], [6].

Seizures can often be the presenting symptom in patients with brain tumors, whether primary or metastatic and whether intraaxial or extraaxial [7]. In some cases, seizures occur even before the tumor is sufficiently established to be correctly identified on computed tomography and magnetic resonance imaging [8]. In patients presenting with other different neurological sequelae, seizures may occur after the diagnosis has been made and, although less likely, even after treatment with surgery or adjuvant therapy [9], [10]. The probability that seizures will be associated with a CNS tumor depends upon the tumor type and grade and its location within the brain or, if extraaxial, its location within the cranial vault [11].

The mechanism behind TAE is likely to be multifactorial, and a number of hypotheses have been proposed. Recent work has explored the role of changes in peritumoral tissue in seizure generation. This has revealed metabolic and pH changes, alterations in levels of neurotransmitters and their receptors, and disruption of localized neural networks in the region of brain tissue surrounding the tumor. This review discusses the pathophysiology behind TAE, the factors affecting the frequency and type of the seizures, and the available treatments and their efficacy.

## 2 Methods

A literature search was performed in MEDLINE through Web of Knowledge (Thomson Reuters) searching for publications between 1990 and 2014. Search criteria were the keywords peritumoral + epilepsy. This search yielded 70 results, 9 of which were review articles. The authors then screened the results and excluded 18 papers that were not relevant before identifying further salient published work from the reference lists of the 52 included.

## 3 Factors governing seizure frequency: tumor type

The frequency and type of seizures associated with TAE depend predominantly upon the type of tumor giving rise to the seizures and the location of that tumor within the brain or, in the case of meningiomas, the location within the skull. All types of primary and secondary brain tumors may present with seizures [12], [13], [14]. Even in those patients who do not suffer from seizures prior to tumor surgery, there is the potential for them to develop TAE in the postoperative period, particularly those with meningiomas [5], [15].

Glioneuronal tumors, primarily arising in children and young adults, have the highest seizure rate, with 85%–92% of dysembryoplastic neuroepithelial tumors (DNETs) and 63%–91% of gangliogliomas presenting with seizures [6], [7], [16], [17], [18]. Glioneuronal tumors, as their name suggests, consist of both dysplastic neurons and neoplastic glial cell elements [19]. Within the tumor, hyperexcitable regions of dysplastic neurones develop, and it is thought that this causes their high degree of epileptogenicity [20].

In tumors of glial origin, low-grade gliomas (World Health Organization (WHO) grades I and II) are more likely to be associated with seizures, with recent studies showing that astrocytomas have a seizure rate of 50%–81%, and oligodendrogliomas have a seizure rate of between 46% and 78% [7], [9], [10], [21]. These tumors grow slowly, invading the surrounding tissue causing gliosis and chronic inflammatory changes in peritumoral regions. Evidence of these inflammatory changes is detectable using immunohistochemical staining; a significant increase in reactive astrocytes is found in cortical peritumoral tissue from patients with chronic seizures compared with peritumoral tissue from patients with no seizures [22].

High-grade gliomas such as glioblastoma multiforme (GBM) are generally thought to be less epileptogenic with a reported seizure rate of between 22% and 62% although this may be a reflection of the shorter survival time associated with this tumor type rather than a true lower rate of tumor epileptogenicity; median survival in patients with GBM is approximately 12 months [5], [7], [23], [24]. Although seizures are less frequent in patients with GBM, they are more difficult to treat, as they are more often refractory to medication and can persist after surgery [25]. It is thought that high-grade gliomas give rise to seizures as a result of localized tissue destruction, ischemia, and necrosis [11], [22], [26]. Because of their growth rate, high-grade tumors are also likely to effect epileptogenic changes in the peritumoral region due to mass effect and as a result of local neuronal network disruption [22]. A hypothesis previously put forward is that seizure activity may be linked to hemosiderin deposition after microhemorrhage from friable tumor vessels present in high-grade gliomas [1]. Increased levels of extracellular iron ions (Fe^3 +^) has been shown experimentally to induce paroxysmal epileptiform activity [27]. However, a recent study showed no relationship between seizure frequency and the presence of hemosiderin on histological examination of samples from 20 patients with GBM [25].

Meningiomas are among the least epileptogenic intracranial tumors with a reported seizure rate of between 13% and 26%, which may be due to the fact that they are extraaxial and, therefore, do not infiltrate the brain parenchyma [15], [28].

## 4 Factors governing seizure frequency and semiology: tumor location

Aside from tumor type, the other most important factor in determining its epileptogenicity is its location. Studies and reviews vary in opinion as to whether frontal, temporal, or parietal lobe tumors are most likely to be associated with seizures, but most agree that occipital lobe lesions are the least epileptogenic [7], [11], [22], [29], [30], [31], [32]. In their review of 2342 patients with TAE from mixed tumor types, Hamasaki et al. reported a frontal predominance in tumor location, specifically in cortical regions close to the motor cortex [7]. Michelucci et al. reviewed 100 patients with seizures related to primary brain tumors and found that 60% were located in the frontal lobe, with temporal and then parietal lobes as the next most common locations [31]. When tumors are grouped by type, different tumor histology is more likely to be related to specific brain regions. Glioneuronal tumors are most commonly located in the temporal lobe and cause predominantly complex partial seizures [6], [19], [33]. High-grade gliomas are most likely to involve multiple brain regions but, when confined to single lobes, are found most commonly in the temporal and frontal lobes [34]. High-grade gliomas made up the majority (79%) of a review of patients with primary brain tumors in whom the initial seizures were predominantly tonic–clonic or focal motor [31]. Location is also a factor in the propensity of meningiomas to cause seizures: convexity and parasagittal/parafalcine meningiomas close to the premotor cortex are associated with the greatest seizure rates (28%–40%), with tuberculum sella meningiomas being the least epileptogenic [15], [28], [35].

It may be that tumors in the anterior frontal lobe are in fact as likely to cause epileptic activity as tumors in the posterior frontal lobe and temporal lobe, but that the relative lack of eloquence of the frontal regions means that some of these seizures go undetected. Although less likely, tumors in the occipital lobe can also produce seizure activity, typically producing visual auras before a seizure [7], [36]. Sellar and skull base tumors, including pituitary tumors and craniopharyngiomas, seem to be much less likely to present with seizures, with Deepak et al. showing a seizure rate of only 9% in their series of 64 patients with macroprolactinomas and Karavitaki et al. finding no seizure activity in a case series of 121 patients with craniopharyngioma [37], [38].

## 5 Pathophysiology

The pathophysiological mechanisms that give rise to epileptic activity in brain tumors are likely to be multifactorial. The literature describes a number of hypotheses relating to the biochemical, microstructural, and electrical environment of the peritumoral area that may give rise to epileptogenesis [1], [14], [26], [29], [33], [39], [40], [41], [42]. These include the levels of neurotransmitters and altered expression of their receptors, altered expression of gap junctions and ion channels, localized pH disturbance, and the effects of disruption of the blood–brain barrier [43], [44], [45], [46], [47].

There is evidence to show that different mechanisms predominate in different tumor types. In tumors containing neurons such as glioneuronal tumors, disruption of neuronal function is the most likely mechanism, whether through the development of hyperexcitable regions of dysplastic neurones within the tumor or neuronal immunoreactivity to certain gap junction proteins (see below) [20], [43], [48]. Both of these factors are likely to contribute to the high degree of epileptogenicity displayed by this tumor type, but other mechanisms must be responsible for tumors of exclusively glial origin, with no neuronal component. Recent evidence suggests that slow growing tumors may induce changes in penumbral connectivity, resulting in the development of network architecture with suboptimal functionality and a lower threshold for seizures [49]. In contrast, higher-grade tumors may induce seizures by tissue damage (ischemia, edema, mass effect, and necrosis) [40].

## 6 Neurotransmitters and receptors in the peritumoral zone

Alterations in glutamate neurotransmission form a core part of the pathophysiology of epileptogenesis. This is not surprising given the excitatory nature of this neurotransmitter in the brain and, thus, its depolarizing action on the neuronal membrane potential [50]. The electrical excitability of tumor cells in gliomas was established in vitro in 1996 [51]. Previously, it had been thought that tumors arising from glial cells, unlike those from neurones, lacked the Na^+^ channels that allow a membrane potential to be generated. However, Patt et al. discovered that a large number of cells in gliomas expressed Na^+^ channels in sufficient quantities to allow generation of brief bursts of action potentials. Activation of glutamate (AMPA/kainate) receptors in glial tumor cell membranes caused this depolarization both in ex vivo human tumor brain slices and cultured human tumor material. These findings indicate that glioma cells may have electrophysiological properties similar to those of neurons [44]. However, evidence to support that these glia with neuron-like properties exert an epileptogenic effect is not presently available. Indeed, it is unlikely that this is the case given that the seizure foci are more often than not found within the peritumoral zone where there is a lower density of glial cells. Moreover, a lack of correlation of glial Na^+^ current density and its position relative to the seizure foci supports this notion [52].

Aside from intrinsic changes in glial cell behavior, these cells are also known to exert a much wider influence on neuronal networks, and they achieve this in a variety of ways. Astrocytes have been shown to be capable of the release, reuptake, and synthesis of neurotransmitters; the provision of neurotransmitter precursors and lactate; the regulation of pH; and the regulation of extracellular K^+^ concentration. Because of the excitatory nature of glutamate, early studies focused on the role of extracellular glutamate in the generation of tumor-associated epilepsy. These findings have not been particularly robust. Bateman et al. demonstrated similar levels of glutamate both in human samples obtained from patients with tumor-associated seizures and patients with tumors who did not display seizures [53]. This study did observe increased glutamine in glioma samples associated with epilepsy. More recently, using microdialysis techniques in patients with high-grade gliomas, we have reported that glutamate is found in higher concentrations in the tumor margin as compared with peritumoral regions [54]. However, it has also been demonstrated that extracellular glutamate is significantly decreased in the peritumoral region of high-grade gliomas [55]. An increase in extracellular glutamate is supported by a study performed by Rosati et al. in 2009. They demonstrated that levels of glutamine synthetase (an enzyme that protects neurons against excitotoxicity by facilitating glutamate uptake and conversion to glutamine) were significantly lower in patients with GBM and epilepsy than in those with nonepileptic GBM [25]. Work by Yuen et al. also implicates glutamate in epileptogenesis; they analyzed a retrospective patient group with samples stored after surgery and a prospective cohort in whom glioma tumoral and peritumoral samples were subsequently taken and found an increased concentration of glutamate in tumor and peritumor which showed a significant correlation with the presence of seizures [56]. They also discovered that expression of the transporter protein system x_c_^−^ was increased in peritumoral tissue and was an independent predictor of the presence of tumor-associated epilepsy. More recently, Buckingham et al. have demonstrated, using in vitro electrophysiology, significant glutamate release in a mouse model in which human derived glial tumors were implanted. Furthermore, this group has shown that the resultant epileptiform activity spreads from glial tumor cells into adjacent brain tissue [57]. The same group has subsequently shown a reduction in the duration of peritumoral epileptiform discharges after the suppression of glutamate release by blocking the transporter protein system x_c_^−^ with sulfasalazine and has concluded that increased extracellular glutamate is, therefore, a causative factor in peritumoral cortical synaptic network hyperexcitability [58]. However, a degree of care should be taken with respect to these findings. Firstly, the authors did not observe spontaneous epileptic activity in peritumoral slices and resorted to the use of the zero magnesium technique to induce epileptic activity in the brain slices obtained from the mouse tumor model. Secondly, while sulfasalazine reduced the duration of interictal epileptiform discharges, it also, paradoxically, increased the rate of these events. Finally, robust concentrations (250 μM) of sulfasalazine failed to completely block the epileptic discharges (measured both at the single neuron and network level) induced by zero magnesium containing artificial cerebrospinal fluid. Moreover, preliminary data from our own work (Cowie and Cunningham, unpublished observations) suggest that much lower concentrations of sulfasalazine (10–20 μM) can completely abolish spontaneous epileptic discharges in human peritumoral slices maintained ex vivo.

In contrast to glutamate, gamma-aminobutyric acid (GABA) is an inhibitory neurotransmitter in the brain and, as such, acts as a counterbalance to excitation. An asymmetry of enhanced excitation and disrupted inhibition has been postulated in a number of epileptic conditions. However, this view has been complicated by recent findings in which *depolarizing* GABA has been described in epileptic human tissue [59], [60]. Thus, for human focal epilepsies, alterations in chloride homeostasis can switch GABA neurotransmission from hyperpolarizing to depolarizing and, therefore, decrease the threshold for seizure genesis. In addition to this finding, a suppression or loss of GABA-mediated inhibition has also been implicated in the pathophysiology of epilepsy, including in peritumoral epilepsy. Using human neurosurgical samples removed from temporal lobe low-grade gliomas, a reduction in somatostatin and GABA immunoreactive neurons has been observed in epileptic peritumoral regions (assessed with intraoperative electrocorticography) as compared with nonepileptic areas surrounding the tumor [61]. Furthermore, a single patient study of the peritumoral neocortex from a temporal lobe astrocytoma revealed a reduction in parvalbumin immunoreactivity and inhibitory synapses at the perisomata and axonal initial segment of pyramidal cells [62]. From a functional perspective, GABA-evoked currents in oocytes injected with membranes obtained from human epileptic peritumoral cerebral cortex show a more depolarized reversal potential when compared with those obtained from nonepileptic healthy cortex, and this difference is attributed to increased expression of two Cl^−^ ion membrane transporters (NKCC1 and KCC2) in neurons in the peritumoral neocortex [63]. This increase in neuronal Cl^−^ transporters will, as in the case of focal epilepsies [59], [60], ensure that GABA acts to depolarize rather than hyperpolarize pyramidal neurons, thereby contributing to epileptogenesis in human brain tumors. Scant evidence exists for concentrations of GABA in peritumoral regions. One study has demonstrated increased GABA in the peritumoral zone of glioblastomas but no similar increase of the inhibitory neurotransmitter in regions surrounding anaplastic astrocytomas and oligodendrogliomas [55].

In addition, functional GABA receptors have been identified on glioma cells themselves. Labrakakis et al. showed the presence of GABA_A_ receptors on WHO grade II and III cells from low-grade gliomas and anaplastic astrocytomas, which, in the majority of their experiments, caused *depolarization* and not hyperpolarization of the glioma cells [45]. Interestingly, the authors demonstrated that application of GABA triggered the activation of voltage-gated Ca^++^ channels and, therefore, hypothesized that the observed depolarization was linked to Ca^++^ influx into the cells. They also showed that these functional GABA receptors were not found in glioblastoma cells, allowing their presence to be used as a marker to differentiate between the tumor types. This finding fits with the known increased epileptogenicity of low-grade gliomas when compared with GBM [7].

Control of extracellular ionic homeostasis is also critical in the genesis of seizures [64]. Central to this is the ability to regulate concentrations of extracellular potassium (K^+^). Extracellular K^+^ homeostasis is conducted by astrocytes by buffering via the Kir4.1 channel. Polymorphisms or mutations of murine and human KCNJ10, which encodes the astroglial Kir4.1 K^+^ channel, are associated with epilepsy [65]. Furthermore, the ability for potassium buffering is impaired in gliomas through a reduction in the expression of Kir4.1 in the plasma membrane of glioma cells [66]. However, at present, no direct evidence exists to demonstrate alterations of Kir4.1 in the peritumoral region and if any change might directly contribute to epileptogenesis in these zones.

## 7 Alterations in pH in the peritumoral zone

In addition to changes in the balance of neurotransmitters, alterations in peritumoral zone pH have also been put forward as a hypothesis for increased epileptogenicity. In general, alkalosis increases membrane excitability, often to an epileptogenic level, while acidosis decreases membrane excitability [67]. However, this is not always the case, and it seems more likely that it is the pH shift in the tumor microenvironment, rather than the absolute pH, which is responsible for the effects on the function of neurotransmitter receptors, gap junctions, and ion channels involved in altering cellular excitability [47]. Using MR spectroscopy, it has been shown that gliomas have a highly acidic extracellular pH, while, conversely, their intracellular pH is alkaline [68], [69], [70]. One hypothesis to explain the underlying mechanism behind the highly acidic extracellular matrix found in gliomas may be that of localized hypoxia [32]. Tumors display a disorganized vasculature, which results in unbalanced perfusion, and are dependant on an adequate blood supply for growth. As gliomas increase in size, they can outgrow their vascular supply and, hence, develop areas of hypoxia [71]. This is particularly true of high-grade gliomas where hypoxia and ischemia can lead to necrosis, a finding often observed at surgery and later on in histology. It is known that in the presence of hypoxia, cellular metabolism favors glucose catabolism and results in lactic acid production. The subsequent acidosis leads to glial cell swelling and damage and it is possible that this may lead to increased seizure generation [32], [72]. In lower grade tumors, localized acidity causes astrocytoma cells and malignant glial cells to demonstrate an increased Na^+^ influx and may lead to increased cellular excitability [71], [73].

However, if this were the only mechanism coupling changes in pH to epileptogenicity, then it ought to be the case that the high-grade tumors most susceptible to hypoxia and ischemia would be those most likely to exhibit seizures, and this is clearly not the case. Therefore, it is likely that if the mechanism described above does play a role, then it is contributed to and modified by other pH-mediated pathophysiology.

Another hypothesis linking pH to epileptogenesis involves the increased expression of carbonic anhydrase (CA) IX in gliomas. Carbonic anhydrase (CA) is the enzyme that catalyzes the reaction CO_2_ + H_2_O to HCO_3_^−^ + H^+^, and its action is upregulated in hypoxia [47]. Carbonic anhydrase IX is unusual when compared with other forms of CA as it spans the cellular membrane and its reaction products are separated: H^+^ is moved out of the cell and HCO_3_^−^ is moved back into the cell cytoplasm. Proescholdt et al. have shown that CA IX was overexpressed in every one of 59 glioblastoma samples they analyzed and also found that it was an independent prognostic factor for poor outcome [70]. This may be the mechanism by which the peritumoral extracellular matrix is kept acidotic and the intracellular environment alkalotic, and doing so is likely to increase the excitability of the peritumoral network. One way in which this may occur is via gap junctions (see below). The pH sensor of these channels has been suggested to be located on the cytoplasmic side of the pore [74], thus an intracellular alkalinization will lead to opening of these channels with implications for neuronal and glial network connectivity.

## 8 Gap junction-mediated connectivity

Gap junctions (GJs) are a means of intercellular communication and consist of membrane proteins (connexins) that form a channel from the membrane of one cell to the membrane of a neighboring cell. They allow the transfer of small metabolites (amino acids, glucose, glutathione, and ATP), small signaling molecules, and microRNAs [75], [76], they are sensitive to changes in pH and Ca^++^ concentrations, and they permit the movement of ions from one cell to the other. Thus, GJs can act as a powerful means of synchronizing network behavior either via direct electrical signaling in neurons or by the propagation of glial Ca^++^ waves. In both instances, increased GJ communication will lead to increases in excitability and a hypersynchronous state. A large body of work has focused on the implications of GJ connectivity between neurons in the hippocampus and neocortex. Gap junction-mediated direct electrical coupling between pyramidal cells [77], [78], [79], [80] and GABAergic interneurons [81], [82], [83], [84], [85], [86] in these regions has been well documented. The dendritic localization of GJ coupling between interneurons [87], [88], [89], [90] has been shown to be important for physiological [91], [92], [93] and pathophysiological [94], [95] neuronal rhythmogenesis. In contrast, there remains a degree of controversy surrounding the precise location of GJ coupling between pyramidal neurons. Schmitz et al. have provided confocal imaging evidence of apposition of CA1 pyramidal cell axons that permits the movement of a fluorescent dye from one neuron to another [96]. In addition, this study also provided physiological evidence in the form of spikelets recorded in CA1 pyramidal cells in the absence of chemical synaptic transmission and in response to CA1 axon collateral stimulation. Furthermore, connexin (Cx) 36 expressing GJs between hippocampal mossy fibers has been demonstrated using a combination of transmission electron microscopy and freeze fracture immune-gold replica labeling [97].

In epileptic human neocortex, a network or ‘plexus’ of GJ-coupled pyramidal cell axons has been demonstrated to underlie the generation of high frequency oscillations (HFOs) [98], [99], [100]. High frequency oscillations are implicated in the epileptogenic process and are emerging as a useful clinical tool in terms of seizure focus delineation [101], [102], [103]. Numerous studies have used either preoperative electroencephalography (EEG) or intraoperative electrocorticography (ECoG) for the functional resection of gliomas in order to gain seizure freedom [24], [104]. At present, it remains unclear if functional resection produces a clear degree of seizure control in patients with TAE (see below). The reasons for this are likely to be multifactorial. However, given the specificity of HFOs to locate seizure-onset zone in intractable focal epilepsy [105], it may be reasonable to propose that a similar temporal high resolution approach should be implemented in surgical treatment. Evidence of HFOs has emerged from one study in which intracranial subdural grids have been used. The patient presented with a right temporal oligodendroglioma and intractable epilepsy, and HFOs coincident with ictal slow direct current (DC) shifts were observed in the peritumoral neocortex [106]. Ex vivo studies conducted in the laboratory of one of the authors have observed the occurrence of spontaneous HFOs associated with interictal discharges in peritumoral tissue obtained from patients undergoing elective neurosurgery for the treatment of TAE (Cunningham et al., unpublished observations). Previous findings from this laboratory have supported the role of axoaxonic gap junction coupling in the generation of HFOs in epileptic tissue obtained from patients with temporal lobe epilepsy [98], [99], [100]. The existence of axoaxonic gap junction coupling in epileptic peritumoral tissue still needs to be tested, but the presence of changes in gap junction coupling between glial cells has been well documented.

Two types of gap junction proteins expressed in glial cells have been studied in relation to peritumoral epilepsy: connexin 32 (Cx32), which is known to be expressed in oligodendrocytes, and connexin 43 (Cx43), the predominant gap junction protein in astrocytes. An early study compared cortical tissue samples from patients undergoing resection for epilepsy, with peritumoral cortical tissue both from patients who had TAE and those who had no seizures [107]. The authors showed an elevation in mRNA coding for the gap junction protein Cx43 both in the patients with intractable epilepsy and in the patients with tumor with seizures and lower levels of the same protein in patients with tumor with no associated seizures. A more recent study by Aronica et al. has demonstrated strong labeling of the Cx32 gap junction protein in glioneuronal tumors (both gangliogliomas and DNETs) and also showed increased expression of the Cx43 gap junction protein in reactive astrocytes in epileptic peritumoral cortex undergoing gliosis in patients with low-grade gliomas [43]. They suggest that the greater expression of these gap junction proteins, specifically in peritumoral astrocytes and in low-grade tumors known to be highly epileptogenic, may mean that they are implicated in synchronization (via astrocytic Ca^++^ elevation) of localized peritumoral neural networks, leading to propagation of seizure activity [108].

## 9 Blood–brain barrier

The blood–brain barrier (BBB) is the means by which the brain cells and their extracellular fluid are kept apart from the blood and protected from harmful molecules within it. It is a function of occluding junctions between capillary epithelial cells, astrocytes, and pericytes and is unique to the capillaries in the central nervous system [32]. Brain tumors are known to cause disruption to the BBB resulting in edema and leaking of microscopic particles and large molecules into the peritumoral region, such as glutamate and albumin, both of which are known to cause seizures [46], [109]. In 2004, Seiffert et al. demonstrated that disruption of the BBB caused foci of epileptiform discharges in rat cortex [110]. More recently, the same group demonstrated delayed neuronal functional impairment following the development of the epileptic focus as a result of BBB disruption [111]. Interestingly, in 2008, Savaskan et al. showed that system x_c_^−^, the glutamate transporter protein overexpressed in gliomas and possibly already implicated in tumor-associated epilepsy (see above), has been shown to be involved in disruption of the blood–brain barrier; animals implanted with a type of system x_c_^−^ with a silenced active subunit had significantly less peritumoral edema compared with animals with normal system x_c_^−^ [112]. The BBB also plays a role in maintaining pH homeostasis through its acid–base transporter molecules, and disruption of the BBB has been shown to increase extracellular pH within the brain [47], [113]. As discussed above, shifts in pH are associated with increased seizure activity, and this mechanism may further implicate BBB disruption in epileptogenesis in the peritumoral zone.

## 10 Treatment of tumor-associated epilepsy

The natural course of epilepsy associated with brain tumors differs from that of epilepsy from other causes. In part this is due to the fact that, in a large number of cases, the patient will undergo surgery for diagnostic, debulking, or curative purposes, and the surgery itself may act to cure the epilepsy. The patient may also undergo adjuvant therapy which can affect seizure frequency or, in the case of chemotherapy, may interact with antiepileptic medication [114]. Most patients with tumor presenting with seizures will be started on an antiepileptic drug (AED), which may be weaned after surgery in the absence of postoperative seizures.

It was previously thought that TAE responded very poorly to antiepileptic medication, but recent studies have demonstrated more encouraging rates of seizure control with newer AEDs [115], [116], [117], [118]. However, in a large number of cases, epilepsy associated with brain tumors can be difficult to manage. Patients with seizures refractory to pharmacological treatment often end up on multiple AED regimes, and those who undergo surgery may be left with seizures postoperatively and still require AEDs [9], [119]. As discussed above, the initial presenting symptom in patients with tumor is often seizures, and they may be started on an AED before a diagnosis is made. Interestingly, as the focus of initial treatment is primarily neurooncological, it is possible that patients with TAE may not be treated by an epileptologist but rather have AEDs started by their emergency physician, neurosurgeon, or neurooncologist. While this management may be adequate, TAE is known to be difficult to control, and, therefore, it would seem most sensible for them to be referred to an epilepsy specialist early in their treatment.

## 11 AEDs

In patients with brain tumor presenting without seizures, the question of whether to initiate *prophylactic* AEDs had previously been based only on an outdated review of the use of prophylactic AEDs in traumatic brain injury [42]. In 2006, a review of the existing literature on the subject was conducted by Perry et al. revealing only a handful of randomized controlled trials, a review, and a single published guideline [120]. The conclusion of their review was that there was no evidence supporting the use of prophylactic AEDs in patients with tumor, as there is no difference in the risk of having a seizure between patients with tumor taking AEDs and those not doing so [121], [122], [123]. Interestingly, a general review of epilepsy in brain tumors in 2010 suggests that as some newer AEDs have an improved safety profile compared with older AEDs, they *should* now be considered for prophylaxis in patients undergoing craniectomy (presumably craniotomy) for tumor, but that they should be stopped one week postoperatively [42]. The same review also states that, in cases where prophylactic AEDs are not given, they should be initiated after only one seizure and that doing so has been shown to reduce the rate of seizure conversion from focal to generalized [124].

Antiepileptic drugs are typically classed into three groups: first-generation drugs, e.g., sodium valproate, phenytoin, carbamazepine, and benzodiazepines; second-generation drugs, e.g., levetiracetam, gabapentin, lamotrigine, and oxcarbazepine; and the newest third-generation drugs, e.g., pregabalin, brivaracetam, and lacosamide [125], [126].

Most AEDs affect neurotransmission by modulating voltage-gated sodium, calcium, or potassium ion channels and/or by action on GABA receptors or by altering GABA concentration, thereby increasing the inhibitory action it has on action potential propagation [127]. A small number of AEDs instead alter glutamate receptors, causing restriction of excitatory neurotransmission.

A recent very thorough review by de Groot et al. lists the studies, both retrospective and prospective, which assess the efficacy of different AEDs specifically in tumor-related epilepsy [40]. All three generations of AEDs have been studied in TAE, but the majority of work has involved more recent drugs such as levetiracetam, topiramate, and gabapentin and has demonstrated rates of seizure freedom with monotherapy at between 20% and 55%, irrespective of the agent studied [128], [129], [130]. However, a retrospective analysis of pediatric patients with TAE showed that those started on second-generation AEDs were less likely to have had to change to a different drug compared with those started on first-generation agents [131]. While the results of all these studies indicate that most AEDs are able to control TAE in up to half the numbers of cases, the published works have suffered from low patient numbers, with the largest group studied numbering only 47 patients [128].

There have also been a small number of papers comparing the use of older AEDs with newer ones, with authors comparing levetiracetam and oxcarbazepine with phenytoin, sodium valproate, and other older AEDs [40], [117], [119], [132]. The results of these predominantly retrospective studies have shown little difference in seizure reduction or freedom between first-generation and second-generation AEDs, but some do show that the newer drugs give rise to fewer side effects [132]. Although it would seem from the research comparing the different types of AEDs that no one drug affords better seizure control, there are drugs which are preferable in TAE due to their reduced effect on the hepatic enzyme cytochrome P450 (CYP) [133]. Older drugs, such as carbamazepine, phenytoin, phenobarbital, and sodium valproate have been found to be either inducers or, in the latter's case, inhibitors of CYP, and this may have implications for patients with tumor having chemotherapy, as some cytotoxic drugs are known to be metabolized by this enzyme [114], [134]. Conversely, it is known that some chemotherapeutic agents are themselves inducers or inhibitors of CYP [135]. Therefore, when patients are treated concurrently with older AEDs and cytotoxic drug therapy, an increase or decrease in plasma concentration of the anticancer drug or AED could occur, resulting in toxicity (with either drug type) or in inadequate chemotherapeutic coverage or loss of seizure control. Interestingly, a review in 2010 by Rossetti and Stupp comments on data from an analysis of the European Organisation for Research and Treatment of Cancer database [42]. It shows that patients with tumor on newer AEDs that do not induce CYP, such as gabapentin, levetiracetam and pregabalin, have a better outcome from chemotherapy. Therefore, AED selection must be considered carefully in patients with TAE who are to undergo chemotherapy to avoid drug interactions and to optimize outcome.

## 12 Surgery

The majority of patients with TAE will undergo surgery at some point in their treatment. Over recent years, particularly in low-grade gliomas, the surgical trend has been to move away from biopsies or limited resections towards maximal safe resection, with evidence showing that this more aggressive approach is associated with better prognosis [48]. Tumor excision has been found to have varying success in the treatment of TAE, with outcome dependent on tumor type, on the severity and frequency of preoperative seizures, and on the degree of surgical resection. This question of surgical resection is possibly the most important factor in tackling TAE, as it is the one factor that can be altered. It seems that the most likely reason for partial resection resulting in poorer control of TAE postoperatively is that the epileptogenic focus has not been removed. It is, therefore, not surprising given the recent move to safely resect more tissue to see that postoperative seizure rates have improved towards the end of the last decade. A retrospective analysis published in 2001 looking only at postsurgical seizure outcome in 45 glioneuronal tumors found that only 63% of patients with gangliogliomas and 58% of those with DNETs were seizure-free after resection [6]. The authors found that young age, total resection, absence of generalized seizures, and shorter duration of epilepsy were predictors of better postsurgical seizure outcome. In 2003, Klein et al. found that 50% of 156 patients with low-grade tumors demonstrated seizures after resective surgery [2]. Hildebrand et al. published their findings in 2005 on 234 patients with gliomas and found that almost 80% experienced tumor-related epilepsy after surgery, although their paper does not state whether the patients simply had biopsies performed for histological diagnosis or whether they had resections or debulking surgeries instead [124]. More recent results have been more encouraging; Babini et al. published the results of their study in 2013 where they compared postoperative seizure rate in children between one group who underwent simple resection for their low-grade glioma and another group who underwent tumor resection along with resection of the surrounding epileptogenic peritumoral zone defined preoperatively using EEG [136]. The seizure-free rate was 80% in those who underwent straightforward resection and 100% in those in whom the resection was extended to include the peritumoral region. Chaichana et al. performed a similar retrospective analysis in 2009 looking at 153 patients operated upon for GBM or anaplastic astrocytoma and found that 77% were seizure-free at 12 months, with lower grade and temporal and/or cortical location as predictors of increased postoperative seizure activity [137]. The same authors performed a further retrospective analysis in 2013, this time looking at seizure outcome after meningioma resection [28]. Of their 84 patients with preoperative seizures, 90% were seizure-free after tumor removal, and they found that the presence of uncontrolled preoperative seizures along with a tumor located on the sphenoid wing or in the parasellar region was an independent predictor of poorer postoperative seizure control.

Neurosurgery for tumor excision where a cure cannot be affected is a balance; in high-grade tumors a greater extent of resection increases time to tumor progression and median survival, and a similar trend suggests that the same may be true of low-grade gliomas [138], [139]. However, the need to remove as much tumor as possible must be countered by the desire to increase *quality* survival and not increased survival at any cost. This aim is achieved at surgery by preserving as much normal brain parenchyma as possible, and the more eloquent the brain area, the more cautious and limited the tumor resection. This conservative approach increases the likelihood that patients suffer from fewer permanent neurological deficits postoperatively but may be implicated in poor postoperative seizure control, especially if resection spares an epileptogenic focus located in the peritumoral region. Where the quality of survival is the main concern, the impact of seizures and the side effect of AEDs must also be considered alongside the need to preserve neurological function.

## 13 Future directions for improved seizure outcome

As mentioned above, there has been a trend in neurooncological surgery towards maximal safe resection. Surgical technology has played a significant part in the safety of this observed increase in the extent of tumor resection and subsequent improvement in postoperative TAE rates. There have been advances in image guidance (both CT and MR), and these have also benefitted from the ability to combine standard anatomical images with information gleaned from functional and diffusion-weighted advanced MR sequences [140], [141]. Initial research on fluorescein-assisted glioma surgery has also shown an increase in the ability to achieve gross total resection over partial resection [142]. The logical next step in glioma surgery must be to enhance resection further not only to improve prognosis but also to improve quality of life by reducing postoperative seizure rates. As more is learned about the neurobiological alterations that occur in the peritumoral zone and their impact on epileptogenesis, it seems more likely that removal of this border of tissue around brain tumors may improve the outcome from TAE. There is already an armamentarium of tests used to locate epileptic foci in surgery for nontumoral epilepsy. These include, but are not limited to, preoperative investigations (EEG, PET scanning, and MR spectroscopy) and intraoperative electrocorticography. Using these techniques, a greater insight into the clinical reliability of a biomarker or a combination of biomarkers (neurotransmitter levels (glutamate, GABA), HFOs, pH) in relation to the peritumoral zone and postsurgical outcomes can be assessed. It seems reasonable to investigate how these modalities and potential biomarker signals obtained with such measurements could be linked to existing image guidance systems to allow increasingly accurate and safe resection of peritumoral tissue for a radical improvement of seizure outcome in TAE.

## Disclosure

MOC and CJAC declare that they have no conflict of interest with respect to the work submitted in this article.
